# Can a registered trial be reported as a one-group, pretest-posttest study with no explanation? A critique of Williams et al. (2021)

**DOI:** 10.1186/s40352-021-00165-3

**Published:** 2022-01-03

**Authors:** Dennis M. Gorman

**Affiliations:** Department of Epidemiology & Biostatistics, School of Public Health, Texas A&M University, Adriance Lab Road, College Station, TX 77843-1266 USA

Williams et al. (2021) assessed the effects of a revised version of the Life Skills Training (LST) program in a juvenile justice diversionary setting. They concluded: “The results indicate that the intervention was effective for a variety of key outcomes” and that the program “can help overcome some of the shortcomings of current youth court programming in a way that is feasible and effective” (Williams et al., 2021, page 9).

There are a number of reasons why the interpretation of the results presented by Williams et al. (2021) in terms of program effectiveness, and their possible impact on youth in the juvenile justice system, are invalid. First, Williams et al. (2021) state “the study design did not include a control or comparison group” (page 10), yet it began as a randomized trial with a control group, as evident from a protocol registered in ClinicalTrials.gov (Williams, 2016). The registry shows the control group was removed from the protocol on January 3, 2019, five months after the study ended. It would be useful to know if the control group data had actually been collected and if the change in study design occurred before or after these data were analyzed. BMC requires registration of all trials and that registration number and date be included as the last line of the manuscript abstract (BMC, 2021). This information does not appear in Williams et al. (2021).

Second, the specific knowledge, attitudes and skills outcomes reported in Williams et al. (2021) are not those described in the registration. The registered primary outcomes were Substance Use Behavior (frequency of use of 13 drugs), Intentions, Attitudes and Perceived Norms, and the secondary outcomes Aggressive, Violent and Delinquent Behavior (frequency of these behaviors). Specific scales are named, such as the Reactive/Proactive Aggression Scale and Rochester Youth Development Self-Reported Delinquency Scale. School performance was also mentioned as an outcome in the “Detailed Description” section of the ClinicalTrials.gov registration. The registered follow-ups were pretest, posttest and 6-month; there is no mention of the latter in Williams et al. (2021). Trial registration is intended to limit selective outcome reporting in publications; this cannot be ruled out in the case of Williams et al. (2021). Concern about such bias has been raised regarding earlier evaluations of the LST program (Brown, 2001; Gorman, 2005a,2005b).

Without a control group one cannot even attribute the pretest-posttest differences in the variables reported in Williams et al. (2021) to participation in the modified version of the LST program. This weak design is further threatened by selection bias as parents of eligible children could decline their participation in the study, but the authors present no data on the number who refused and how they differed from those whose parents consented to their involvement. They do, in fact, acknowledge the inability of their one-group, pretest-posttest study design to rule out competing explanations of change. Yet they persist in claiming: “The findings indicate that an evidence-based prevention approach adapted for youth diversionary settings … can produce positive changes in psychosocial skills and protective factors known to prevent multiple risk behaviors among youth” (Williams et al., 2021, page 1). The registered study, with a control group, prespecified behavioral outcomes and six-month follow-up could have addressed the effectiveness of the LST program in the juvenile justice arena. The modified study reported in Williams et al. (2021) cannot and may be subject to selective outcome reporting bias.

## Data Availability

The data discussed are in the paper that is the focus of the critique and the ClinicalTrials.gov webpage.
